# *trans*-Dichloridobis(triphenyl­phosphane-κ*P*)palladium(II) benzene hemisolvate

**DOI:** 10.1107/S1600536812012494

**Published:** 2012-03-31

**Authors:** Frank Meyer-Wegner, Hans-Wolfram Lerner, Tanja Sinke, Michael Bolte

**Affiliations:** a Institut für Anorganische und Analytische Chemie, Goethe-Universität Frankfurt, Max-von-Laue-Strasse 7, 60438 Frankfurt am Main, Germany

## Abstract

The title complex, [PdCl_2_(C_18_H_15_P)_2_]·0.5C_6_H_6_, has the Pd^II^ ion in a square-planar coordination mode (r.m.s. deviation for Pd, P and Cl atoms = 0.024 Å) with the PPh_3_ and Cl ligands mutually *trans*. The benzene solvent mol­ecule is located about a crystallographic inversion centre. The title complex is isostructural with *trans*-dichloridobis(triphenyl­phosphane)­palladium(II) 1,4-dichloro­benzene sesquisolvate [Kitano *et al.* (1983 ▶). *Acta Cryst.* C**39**, 1015–1017].

## Related literature

For the synthetic background, see: Lerner (2005 ▶); Meyer-Wegner *et al.* (2009 ▶, 2011 ▶). For *trans*-dichlorido-bis­(triphenyl­phosphane)palladium(II) sesqui(*p*-dichloro­benzene), see: Kitano *et al.* (1983 ▶).
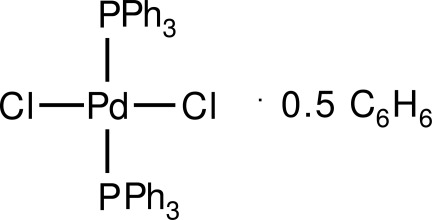


## Experimental

### 

#### Crystal data

[PdCl_2_(C_18_H_15_P)_2_]·0.5C_6_H_6_
*M* *_r_* = 740.89
Monoclinic, 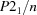

*a* = 11.4530 (7) Å
*b* = 18.4493 (8) Å
*c* = 16.4696 (10) Å
β = 104.979 (5)°
*V* = 3361.8 (3) Å^3^
*Z* = 4
Mo *K*α radiation
μ = 0.83 mm^−1^
*T* = 173 K
0.26 × 0.08 × 0.08 mm

#### Data collection

Stoe IPDS II two-circle diffractometer
Absorption correction: multi-scan (*X-AREA*; Stoe & Cie, 2001 ▶) *T*_min_ = 0.812, *T*_max_ = 0.936
42186 measured reflections
6302 independent reflections
5104 reflections with *I* > 2σ(*I*)
*R*_int_ = 0.089

#### Refinement

*R*[*F* ^2^ > 2σ(*F* ^2^)] = 0.041
*wR*(*F* ^2^) = 0.095
*S* = 1.03
6302 reflections
397 parameters
4 restraints
H-atom parameters constrained
Δρ_max_ = 0.45 e Å^−3^
Δρ_min_ = −0.76 e Å^−3^

### 

Data collection: *X-AREA* (Stoe & Cie, 2001 ▶); cell refinement: *X-AREA*; data reduction: *X-AREA*; program(s) used to solve structure: *SHELXS97* (Sheldrick, 2008 ▶); program(s) used to refine structure: *SHELXL97* (Sheldrick, 2008 ▶); molecular graphics: *XP* (Sheldrick, 2008 ▶); software used to prepare material for publication: *SHELXL97* and *PLATON* (Spek, 2009 ▶).

## Supplementary Material

Crystal structure: contains datablock(s) I, global. DOI: 10.1107/S1600536812012494/tk5074sup1.cif

Structure factors: contains datablock(s) I. DOI: 10.1107/S1600536812012494/tk5074Isup2.hkl

Additional supplementary materials:  crystallographic information; 3D view; checkCIF report

## Figures and Tables

**Fig. 1.**
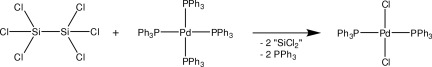
Reaction scheme.

**Fig. 2.**
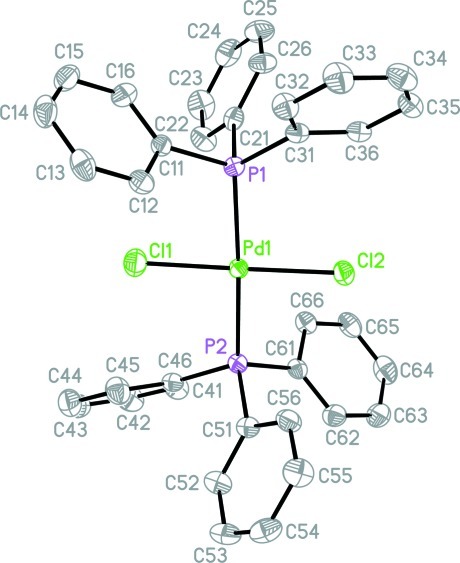
A perspective view of the title compound, showing the atom-numbering scheme. Displacement ellipsoids are drawn at the 50% probability level. H atoms and the benzene molecule are omitted for clarity.

**Fig. 3.**
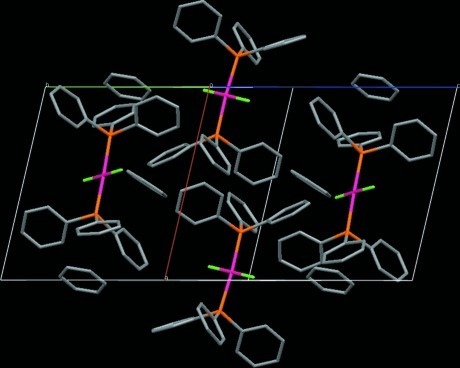
Packing diagram of the title compound. H atoms omitted for clarity.

## supplementary crystallographic information

### Comment

Recently, we have shown that the degradation of Cl_3_SiSiCl_3_ in the presence of donors, such as amines N*R*_3_ (*R* = Me, Et) and phosphanes P*R*_3_ (*R* = *t*Bu) and the isoelectronic silanide [Si*t*Bu_3_]^-^ (Lerner, 2005), in the first step gives dichlorsilylene SiCl_2_ and ultimately produces the perchlorinated neopentasilane Si(SiCl_3_)_4_ and in the case of the silanide the silatetrahedrane (*t*Bu_3_Si)_4_Si_4_, respectively (Meyer-Wegner *et al.*, 2009, 2011). Moreover, we verified that the donor-induced degradation of Cl_3_SiSiCl_3_ in the presence of the silylene trapping agent 2,3-dimethyl-1,3-butadiene gives the [4 + 1] cycloadduct (Meyer-Wegner *et al.*, 2011). We are currently interested in dichlorsilylene transition metal complexes. To this end, we thought that such complexes can be prepared using the reaction between Cl_3_SiSiCl_3_ and [Pd(PPh_3_)_4_]. However, the reaction of Cl_3_SiSiCl_3_ with [Pd(PPh_3_)_4_] gives exclusively [PdCl_2_(PPh_3_)_2_] (Fig. 1). Apparently no dichlorsilylene transition metal complex was formed thereby. Single crystals composed of one have molecule of benzene and one molecule of [PdCl_2_(PPh_3_)_2_] could be isolated from the reaction solution.

The title complex (Fig. 2) has the Pd centre in a quadratic planar (r.m.s. deviation for Pd, P and Cl atoms: 0.024 Å) coordination mode with the PPh_3_ and Cl ligands mutually *trans*. The solvent benzene molecule is located about a crystallographic inversion centre. The title compound is isomorphous with *trans*-dichlorido-bis(triphenylphosphane)palladium(II) sesqui(*p*-dichlorobenzene) (Kitano *et al.*, 1983). The packing diagram (Fig. 3) shows how the benzene molecules fill the empty space between the complexes. There are no unusual features.

### Experimental

Pd(PPh_3_)_4_ (0.19 g, 0.16 mmol) was suspended in benzene (5 ml) and then treated with one equivalent of Si_2_Cl_6_ (0.028 ml, 44 mg, 0.16 mmol) in benzene (0.5 ml) at ambient temperature. The solution immediately became clear and turned from yellow to dark red. Crystals of the title compound were obtained by slow evaporation of the solvent at ambient temperature.

### Refinement

H atoms were refined using a riding model, with C—H = 0.95 Å and with *U*_iso_(H) = 1.2*U*_eq_(C). The C—C distances in the solvent benzene molecule were restrained to 1.39 (1) Å.

### Crystal data

**Table:**

[PdCl_2_(C_18_H_15_P)_2_]·0.5C_6_H_6_	*F*(000) = 1508
*M**_r_* = 740.89	*D*_x_ = 1.464 Mg m^−^^3^
Monoclinic, *P*2_1_/*n*	Mo *K*α radiation, λ = 0.71073 Å
Hall symbol: -P 2yn	Cell parameters from 28523 reflections
*a* = 11.4530 (7) Å	θ = 3.3–26.1°
*b* = 18.4493 (8) Å	µ = 0.83 mm^−^^1^
*c* = 16.4696 (10) Å	*T* = 173 K
β = 104.979 (5)°	Rod, yellow
*V* = 3361.8 (3) Å^3^	0.26 × 0.08 × 0.08 mm
*Z* = 4	

### Data collection

**Table:**

Stoe IPDS II two-circle diffractometer	6302 independent reflections
Radiation source: Genix 3D IµS microfocus X-ray source	5104 reflections with *I* > 2σ(*I*)
Genix 3D multilayer optics monochromator	*R*_int_ = 0.089
ω scans	θ_max_ = 25.7°, θ_min_ = 3.3°
Absorption correction: multi-scan (*X-AREA*; Stoe & Cie, 2001)	*h* = −13→13
*T*_min_ = 0.812, *T*_max_ = 0.936	*k* = −22→22
42186 measured reflections	*l* = −20→19

### Refinement

**Table:**

Refinement on *F*^2^	Primary atom site location: structure-invariant direct methods
Least-squares matrix: full	Secondary atom site location: difference Fourier map
*R*[*F*^2^ > 2σ(*F*^2^)] = 0.041	Hydrogen site location: inferred from neighbouring sites
*wR*(*F*^2^) = 0.095	H-atom parameters constrained
*S* = 1.03	*w* = 1/[σ^2^(*F*_o_^2^) + (0.0537*P*)^2^] where *P* = (*F*_o_^2^ + 2*F*_c_^2^)/3
6302 reflections	(Δ/σ)_max_ = 0.001
397 parameters	Δρ_max_ = 0.45 e Å^−^^3^
4 restraints	Δρ_min_ = −0.76 e Å^−^^3^

### Special details

**Table:**

Geometry. All e.s.d.'s (except the e.s.d. in the dihedral angle between two l.s. planes) are estimated using the full covariance matrix. The cell e.s.d.'s are taken into account individually in the estimation of e.s.d.'s in distances, angles and torsion angles; correlations between e.s.d.'s in cell parameters are only used when they are defined by crystal symmetry. An approximate (isotropic) treatment of cell e.s.d.'s is used for estimating e.s.d.'s involving l.s. planes.
Refinement. Refinement of *F*^2^ against ALL reflections. The weighted *R*-factor *wR* and goodness of fit *S* are based on *F*^2^, conventional *R*-factors *R* are based on *F*, with *F* set to zero for negative *F*^2^. The threshold expression of *F*^2^ > σ(*F*^2^) is used only for calculating *R*-factors(gt) *etc*. and is not relevant to the choice of reflections for refinement. *R*-factors based on *F*^2^ are statistically about twice as large as those based on *F*, and *R*- factors based on ALL data will be even larger.

### Fractional atomic coordinates and isotropic or equivalent isotropic displacement parameters (Å^2^)

**Table:**

	*x*	*y*	*z*	*U*_iso_*/*U*_eq_	
Pd1	0.45706 (2)	0.831821 (14)	0.206242 (15)	0.01813 (9)	
Cl1	0.42899 (9)	0.70941 (5)	0.19156 (6)	0.0353 (2)	
Cl2	0.48343 (8)	0.95572 (5)	0.21552 (6)	0.0292 (2)	
P1	0.25111 (7)	0.83867 (5)	0.20060 (5)	0.01825 (18)	
P2	0.65875 (7)	0.81933 (5)	0.20311 (5)	0.01921 (18)	
C11	0.1927 (3)	0.75972 (18)	0.2434 (2)	0.0210 (7)	
C12	0.2494 (3)	0.7383 (2)	0.3252 (2)	0.0255 (7)	
H12	0.3193	0.7632	0.3561	0.031*	
C13	0.2041 (4)	0.6805 (2)	0.3619 (2)	0.0320 (9)	
H13	0.2417	0.6669	0.4182	0.038*	
C14	0.1042 (4)	0.6429 (2)	0.3163 (3)	0.0363 (9)	
H14	0.0729	0.6035	0.3413	0.044*	
C15	0.0500 (4)	0.6625 (2)	0.2346 (3)	0.0339 (8)	
H15	−0.0171	0.6357	0.2027	0.041*	
C16	0.0930 (3)	0.7213 (2)	0.1988 (2)	0.0284 (8)	
H16	0.0536	0.7353	0.1430	0.034*	
C21	0.1665 (3)	0.84101 (19)	0.0905 (2)	0.0221 (7)	
C22	0.2071 (4)	0.8002 (2)	0.0328 (2)	0.0307 (8)	
H22	0.2799	0.7733	0.0505	0.037*	
C23	0.1417 (4)	0.7986 (2)	−0.0508 (2)	0.0387 (10)	
H23	0.1690	0.7695	−0.0898	0.046*	
C24	0.0377 (4)	0.8389 (2)	−0.0778 (2)	0.0363 (9)	
H24	−0.0066	0.8378	−0.1351	0.044*	
C25	−0.0017 (4)	0.8810 (3)	−0.0206 (2)	0.0375 (10)	
H25	−0.0724	0.9098	−0.0392	0.045*	
C26	0.0608 (3)	0.8817 (2)	0.0633 (2)	0.0288 (8)	
H26	0.0319	0.9097	0.1024	0.035*	
C31	0.1919 (3)	0.91423 (19)	0.2491 (2)	0.0208 (7)	
C32	0.1352 (3)	0.9029 (2)	0.3138 (2)	0.0276 (8)	
H32	0.1272	0.8553	0.3335	0.033*	
C33	0.0908 (4)	0.9614 (2)	0.3490 (3)	0.0348 (9)	
H33	0.0523	0.9535	0.3929	0.042*	
C34	0.1013 (4)	1.0303 (2)	0.3217 (2)	0.0330 (9)	
H34	0.0702	1.0699	0.3464	0.040*	
C35	0.1580 (3)	1.0425 (2)	0.2574 (2)	0.0282 (8)	
H35	0.1657	1.0905	0.2385	0.034*	
C36	0.2029 (3)	0.98475 (19)	0.2212 (2)	0.0231 (7)	
H36	0.2413	0.9931	0.1773	0.028*	
C41	0.7013 (3)	0.73483 (19)	0.1600 (2)	0.0246 (7)	
C42	0.6964 (4)	0.6693 (2)	0.2010 (3)	0.0361 (9)	
H42	0.6730	0.6691	0.2523	0.043*	
C43	0.7250 (4)	0.6044 (2)	0.1681 (3)	0.0427 (10)	
H43	0.7216	0.5602	0.1970	0.051*	
C44	0.7585 (4)	0.6041 (2)	0.0931 (3)	0.0424 (11)	
H44	0.7772	0.5598	0.0699	0.051*	
C45	0.7642 (4)	0.6682 (3)	0.0528 (3)	0.0424 (10)	
H45	0.7871	0.6681	0.0014	0.051*	
C46	0.7371 (4)	0.7336 (2)	0.0858 (2)	0.0312 (8)	
H46	0.7432	0.7776	0.0574	0.037*	
C51	0.7630 (3)	0.82704 (19)	0.3072 (2)	0.0218 (7)	
C52	0.8674 (3)	0.7857 (2)	0.3317 (2)	0.0285 (8)	
H52	0.8875	0.7529	0.2930	0.034*	
C53	0.9426 (3)	0.7919 (2)	0.4122 (2)	0.0327 (9)	
H53	1.0135	0.7630	0.4284	0.039*	
C54	0.9154 (3)	0.8397 (2)	0.4689 (2)	0.0329 (9)	
H54	0.9670	0.8437	0.5241	0.039*	
C55	0.8125 (4)	0.8818 (2)	0.4449 (2)	0.0349 (9)	
H55	0.7940	0.9152	0.4837	0.042*	
C56	0.7358 (3)	0.8758 (2)	0.3645 (2)	0.0291 (8)	
H56	0.6650	0.9048	0.3486	0.035*	
C61	0.7014 (3)	0.89120 (18)	0.1407 (2)	0.0211 (7)	
C62	0.8040 (3)	0.9323 (2)	0.1700 (2)	0.0288 (8)	
H62	0.8577	0.9215	0.2230	0.035*	
C63	0.8293 (4)	0.9893 (2)	0.1227 (3)	0.0367 (9)	
H63	0.9004	1.0173	0.1433	0.044*	
C64	0.7520 (4)	1.0056 (2)	0.0460 (3)	0.0375 (9)	
H64	0.7696	1.0449	0.0138	0.045*	
C65	0.6488 (4)	0.9648 (2)	0.0157 (2)	0.0318 (8)	
H65	0.5958	0.9759	−0.0374	0.038*	
C66	0.6223 (3)	0.9076 (2)	0.0625 (2)	0.0261 (7)	
H66	0.5511	0.8797	0.0418	0.031*	
C1	0.4356 (5)	1.0112 (4)	0.4195 (3)	0.0687 (19)	
H1	0.3902	1.0194	0.3633	0.082*	
C2	0.4811 (6)	0.9443 (4)	0.4426 (4)	0.077 (2)	
H2	0.4682	0.9059	0.4028	0.093*	
C3	0.5455 (6)	0.9327 (4)	0.5236 (4)	0.080 (2)	
H3	0.5774	0.8859	0.5407	0.096*	

### Atomic displacement parameters (Å^2^)

**Table:**

	*U*^11^	*U*^22^	*U*^33^	*U*^12^	*U*^13^	*U*^23^
Pd1	0.01820 (13)	0.01790 (14)	0.01891 (13)	0.00024 (10)	0.00593 (9)	0.00096 (10)
Cl1	0.0365 (5)	0.0263 (5)	0.0458 (6)	−0.0019 (4)	0.0154 (4)	−0.0002 (4)
Cl2	0.0279 (4)	0.0214 (4)	0.0400 (5)	0.0020 (3)	0.0116 (4)	−0.0015 (4)
P1	0.0186 (4)	0.0204 (4)	0.0166 (4)	−0.0005 (3)	0.0061 (3)	−0.0001 (3)
P2	0.0191 (4)	0.0201 (4)	0.0190 (4)	0.0008 (3)	0.0060 (3)	0.0011 (3)
C11	0.0201 (16)	0.0188 (17)	0.0254 (17)	0.0008 (13)	0.0082 (13)	−0.0007 (13)
C12	0.0251 (18)	0.0266 (19)	0.0256 (18)	0.0041 (14)	0.0079 (14)	−0.0004 (14)
C13	0.043 (2)	0.027 (2)	0.0301 (19)	0.0080 (16)	0.0168 (17)	0.0063 (15)
C14	0.043 (2)	0.024 (2)	0.050 (2)	−0.0011 (17)	0.028 (2)	0.0041 (17)
C15	0.0290 (19)	0.030 (2)	0.045 (2)	−0.0075 (16)	0.0129 (16)	−0.0047 (18)
C16	0.0248 (18)	0.031 (2)	0.0299 (19)	−0.0036 (15)	0.0084 (15)	−0.0018 (15)
C21	0.0277 (18)	0.0216 (18)	0.0184 (16)	−0.0039 (14)	0.0087 (13)	0.0007 (13)
C22	0.035 (2)	0.035 (2)	0.0235 (18)	0.0072 (16)	0.0096 (15)	−0.0008 (16)
C23	0.053 (3)	0.041 (2)	0.0216 (18)	0.001 (2)	0.0084 (17)	−0.0061 (17)
C24	0.043 (2)	0.041 (2)	0.0197 (17)	−0.0112 (19)	−0.0011 (16)	−0.0035 (16)
C25	0.027 (2)	0.048 (3)	0.031 (2)	−0.0006 (17)	−0.0037 (16)	0.0011 (18)
C26	0.0220 (17)	0.041 (2)	0.0227 (17)	0.0002 (15)	0.0040 (14)	−0.0057 (15)
C31	0.0177 (16)	0.0265 (18)	0.0176 (15)	0.0009 (13)	0.0034 (12)	−0.0051 (13)
C32	0.0304 (19)	0.029 (2)	0.0265 (18)	0.0022 (15)	0.0134 (15)	0.0000 (15)
C33	0.039 (2)	0.038 (2)	0.034 (2)	0.0046 (17)	0.0219 (17)	−0.0028 (17)
C34	0.033 (2)	0.039 (2)	0.0275 (19)	0.0101 (17)	0.0094 (16)	−0.0057 (16)
C35	0.0285 (18)	0.027 (2)	0.0283 (18)	0.0056 (15)	0.0057 (15)	0.0017 (15)
C36	0.0189 (16)	0.0287 (19)	0.0216 (16)	0.0020 (13)	0.0052 (13)	0.0003 (14)
C41	0.0194 (17)	0.0253 (19)	0.0270 (18)	0.0019 (13)	0.0018 (14)	−0.0003 (14)
C42	0.037 (2)	0.032 (2)	0.043 (2)	0.0045 (17)	0.0184 (18)	0.0009 (18)
C43	0.039 (2)	0.029 (2)	0.060 (3)	0.0024 (17)	0.014 (2)	0.002 (2)
C44	0.034 (2)	0.037 (2)	0.052 (3)	0.0040 (18)	0.0030 (19)	−0.015 (2)
C45	0.045 (2)	0.048 (3)	0.035 (2)	0.012 (2)	0.0112 (18)	−0.006 (2)
C46	0.035 (2)	0.034 (2)	0.0255 (19)	0.0057 (16)	0.0103 (16)	−0.0032 (15)
C51	0.0206 (16)	0.0272 (18)	0.0189 (15)	−0.0007 (14)	0.0077 (12)	0.0061 (14)
C52	0.0262 (18)	0.037 (2)	0.0238 (17)	0.0055 (15)	0.0090 (14)	0.0043 (15)
C53	0.0216 (18)	0.046 (2)	0.0304 (19)	0.0048 (16)	0.0055 (15)	0.0069 (18)
C54	0.0286 (19)	0.049 (3)	0.0196 (17)	−0.0083 (17)	0.0039 (14)	0.0028 (17)
C55	0.037 (2)	0.042 (2)	0.0264 (19)	0.0038 (18)	0.0099 (16)	−0.0045 (17)
C56	0.0265 (18)	0.029 (2)	0.0285 (18)	0.0058 (15)	0.0019 (15)	0.0000 (15)
C61	0.0208 (16)	0.0213 (17)	0.0238 (16)	0.0032 (13)	0.0108 (13)	−0.0002 (13)
C62	0.0252 (18)	0.032 (2)	0.0302 (19)	−0.0013 (15)	0.0090 (15)	0.0051 (16)
C63	0.030 (2)	0.034 (2)	0.048 (2)	−0.0062 (16)	0.0120 (17)	0.0080 (18)
C64	0.046 (2)	0.032 (2)	0.042 (2)	0.0038 (18)	0.0244 (19)	0.0128 (18)
C65	0.040 (2)	0.031 (2)	0.0256 (18)	0.0090 (17)	0.0109 (16)	0.0078 (15)
C66	0.0294 (19)	0.0261 (19)	0.0233 (17)	0.0034 (14)	0.0075 (14)	0.0012 (14)
C1	0.037 (3)	0.137 (6)	0.029 (2)	−0.005 (3)	0.0023 (19)	0.000 (3)
C2	0.069 (4)	0.113 (6)	0.062 (4)	−0.034 (4)	0.039 (3)	−0.031 (4)
C3	0.069 (4)	0.092 (5)	0.096 (5)	0.016 (4)	0.053 (4)	0.028 (4)

### Geometric parameters (Å, º)

**Table:**

Pd1—Cl1	2.2851 (10)	C36—H36	0.9500
Pd1—Cl2	2.3056 (9)	C41—C46	1.386 (5)
Pd1—P2	2.3351 (9)	C41—C42	1.392 (6)
Pd1—P1	2.3399 (9)	C42—C43	1.388 (6)
P1—C11	1.819 (3)	C42—H42	0.9500
P1—C31	1.822 (3)	C43—C44	1.384 (7)
P1—C21	1.822 (3)	C43—H43	0.9500
P2—C61	1.820 (4)	C44—C45	1.366 (7)
P2—C51	1.824 (3)	C44—H44	0.9500
P2—C41	1.830 (4)	C45—C46	1.391 (6)
C11—C16	1.383 (5)	C45—H45	0.9500
C11—C12	1.394 (5)	C46—H46	0.9500
C12—C13	1.389 (5)	C51—C52	1.387 (5)
C12—H12	0.9500	C51—C56	1.396 (5)
C13—C14	1.383 (6)	C52—C53	1.386 (5)
C13—H13	0.9500	C52—H52	0.9500
C14—C15	1.377 (6)	C53—C54	1.378 (6)
C14—H14	0.9500	C53—H53	0.9500
C15—C16	1.383 (6)	C54—C55	1.381 (6)
C15—H15	0.9500	C54—H54	0.9500
C16—H16	0.9500	C55—C56	1.392 (5)
C21—C22	1.383 (5)	C55—H55	0.9500
C21—C26	1.395 (5)	C56—H56	0.9500
C22—C23	1.388 (5)	C61—C62	1.377 (5)
C22—H22	0.9500	C61—C66	1.402 (5)
C23—C24	1.376 (6)	C62—C63	1.383 (5)
C23—H23	0.9500	C62—H62	0.9500
C24—C25	1.383 (6)	C63—C64	1.375 (6)
C24—H24	0.9500	C63—H63	0.9500
C25—C26	1.383 (5)	C64—C65	1.381 (6)
C25—H25	0.9500	C64—H64	0.9500
C26—H26	0.9500	C65—C66	1.385 (5)
C31—C36	1.396 (5)	C65—H65	0.9500
C31—C32	1.400 (5)	C66—H66	0.9500
C32—C33	1.382 (5)	C1—C3^i^	1.376 (6)
C32—H32	0.9500	C1—C2	1.355 (7)
C33—C34	1.364 (6)	C1—H1	0.9500
C33—H33	0.9500	C2—C3	1.365 (7)
C34—C35	1.396 (6)	C2—H2	0.9500
C34—H34	0.9500	C3—C1^i^	1.376 (6)
C35—C36	1.383 (5)	C3—H3	0.9500
C35—H35	0.9500		
			
Cl1—Pd1—Cl2	177.80 (4)	C34—C35—H35	120.0
Cl1—Pd1—P2	90.75 (3)	C35—C36—C31	120.0 (3)
Cl2—Pd1—P2	89.20 (3)	C35—C36—H36	120.0
Cl1—Pd1—P1	86.35 (3)	C31—C36—H36	120.0
Cl2—Pd1—P1	93.58 (3)	C46—C41—C42	118.1 (4)
P2—Pd1—P1	175.72 (3)	C46—C41—P2	121.7 (3)
C11—P1—C31	103.13 (16)	C42—C41—P2	120.2 (3)
C11—P1—C21	104.49 (16)	C43—C42—C41	121.1 (4)
C31—P1—C21	104.77 (16)	C43—C42—H42	119.5
C11—P1—Pd1	114.15 (11)	C41—C42—H42	119.5
C31—P1—Pd1	120.63 (11)	C44—C43—C42	120.0 (4)
C21—P1—Pd1	108.21 (11)	C44—C43—H43	120.0
C61—P2—C51	105.54 (16)	C42—C43—H43	120.0
C61—P2—C41	105.34 (16)	C45—C44—C43	119.3 (4)
C51—P2—C41	104.81 (16)	C45—C44—H44	120.4
C61—P2—Pd1	110.59 (11)	C43—C44—H44	120.4
C51—P2—Pd1	112.49 (11)	C44—C45—C46	121.2 (4)
C41—P2—Pd1	117.17 (12)	C44—C45—H45	119.4
C16—C11—C12	118.8 (3)	C46—C45—H45	119.4
C16—C11—P1	122.7 (3)	C41—C46—C45	120.4 (4)
C12—C11—P1	118.4 (3)	C41—C46—H46	119.8
C13—C12—C11	120.4 (3)	C45—C46—H46	119.8
C13—C12—H12	119.8	C52—C51—C56	118.9 (3)
C11—C12—H12	119.8	C52—C51—P2	122.4 (3)
C14—C13—C12	119.9 (4)	C56—C51—P2	118.6 (3)
C14—C13—H13	120.0	C53—C52—C51	120.6 (4)
C12—C13—H13	120.0	C53—C52—H52	119.7
C15—C14—C13	119.9 (4)	C51—C52—H52	119.7
C15—C14—H14	120.0	C54—C53—C52	120.5 (4)
C13—C14—H14	120.0	C54—C53—H53	119.8
C14—C15—C16	120.2 (4)	C52—C53—H53	119.8
C14—C15—H15	119.9	C53—C54—C55	119.5 (3)
C16—C15—H15	119.9	C53—C54—H54	120.3
C11—C16—C15	120.8 (4)	C55—C54—H54	120.3
C11—C16—H16	119.6	C54—C55—C56	120.7 (4)
C15—C16—H16	119.6	C54—C55—H55	119.7
C22—C21—C26	119.3 (3)	C56—C55—H55	119.7
C22—C21—P1	118.9 (3)	C55—C56—C51	119.9 (3)
C26—C21—P1	121.8 (3)	C55—C56—H56	120.1
C21—C22—C23	120.2 (4)	C51—C56—H56	120.1
C21—C22—H22	119.9	C62—C61—C66	119.5 (3)
C23—C22—H22	119.9	C62—C61—P2	122.2 (3)
C24—C23—C22	120.6 (4)	C66—C61—P2	118.2 (3)
C24—C23—H23	119.7	C61—C62—C63	120.4 (4)
C22—C23—H23	119.7	C61—C62—H62	119.8
C23—C24—C25	119.4 (3)	C63—C62—H62	119.8
C23—C24—H24	120.3	C64—C63—C62	120.3 (4)
C25—C24—H24	120.3	C64—C63—H63	119.8
C24—C25—C26	120.6 (4)	C62—C63—H63	119.8
C24—C25—H25	119.7	C63—C64—C65	120.0 (4)
C26—C25—H25	119.7	C63—C64—H64	120.0
C25—C26—C21	119.9 (4)	C65—C64—H64	120.0
C25—C26—H26	120.0	C64—C65—C66	120.3 (4)
C21—C26—H26	120.0	C64—C65—H65	119.9
C36—C31—C32	119.2 (3)	C66—C65—H65	119.9
C36—C31—P1	119.5 (3)	C65—C66—C61	119.5 (3)
C32—C31—P1	121.2 (3)	C65—C66—H66	120.2
C33—C32—C31	119.8 (4)	C61—C66—H66	120.2
C33—C32—H32	120.1	C3^i^—C1—C2	120.9 (5)
C31—C32—H32	120.1	C3^i^—C1—H1	119.6
C34—C33—C32	121.0 (4)	C2—C1—H1	119.6
C34—C33—H33	119.5	C1—C2—C3	119.3 (6)
C32—C33—H33	119.5	C1—C2—H2	120.4
C33—C34—C35	119.9 (4)	C3—C2—H2	120.4
C33—C34—H34	120.0	C1^i^—C3—C2	119.9 (6)
C35—C34—H34	120.0	C1^i^—C3—H3	120.1
C36—C35—C34	120.1 (4)	C2—C3—H3	120.1
C36—C35—H35	120.0		
			
Cl1—Pd1—P1—C11	31.06 (13)	C32—C33—C34—C35	−0.2 (6)
Cl2—Pd1—P1—C11	−151.14 (12)	C33—C34—C35—C36	0.3 (6)
Cl1—Pd1—P1—C31	154.76 (13)	C34—C35—C36—C31	−0.2 (5)
Cl2—Pd1—P1—C31	−27.44 (13)	C32—C31—C36—C35	0.0 (5)
Cl1—Pd1—P1—C21	−84.79 (12)	P1—C31—C36—C35	179.8 (3)
Cl2—Pd1—P1—C21	93.00 (12)	C61—P2—C41—C46	−10.0 (3)
Cl1—Pd1—P2—C61	139.03 (12)	C51—P2—C41—C46	−121.1 (3)
Cl2—Pd1—P2—C61	−38.77 (12)	Pd1—P2—C41—C46	113.4 (3)
Cl1—Pd1—P2—C51	−103.24 (13)	C61—P2—C41—C42	171.1 (3)
Cl2—Pd1—P2—C51	78.96 (13)	C51—P2—C41—C42	60.0 (3)
Cl1—Pd1—P2—C41	18.33 (13)	Pd1—P2—C41—C42	−65.5 (3)
Cl2—Pd1—P2—C41	−159.48 (13)	C46—C41—C42—C43	−0.8 (6)
C31—P1—C11—C16	100.7 (3)	P2—C41—C42—C43	178.2 (3)
C21—P1—C11—C16	−8.6 (3)	C41—C42—C43—C44	−0.4 (7)
Pd1—P1—C11—C16	−126.6 (3)	C42—C43—C44—C45	0.7 (7)
C31—P1—C11—C12	−77.8 (3)	C43—C44—C45—C46	0.0 (7)
C21—P1—C11—C12	172.9 (3)	C42—C41—C46—C45	1.6 (6)
Pd1—P1—C11—C12	54.9 (3)	P2—C41—C46—C45	−177.4 (3)
C16—C11—C12—C13	−1.8 (5)	C44—C45—C46—C41	−1.2 (6)
P1—C11—C12—C13	176.7 (3)	C61—P2—C51—C52	−96.0 (3)
C11—C12—C13—C14	1.7 (6)	C41—P2—C51—C52	15.0 (3)
C12—C13—C14—C15	0.2 (6)	Pd1—P2—C51—C52	143.4 (3)
C13—C14—C15—C16	−2.0 (6)	C61—P2—C51—C56	84.8 (3)
C12—C11—C16—C15	0.0 (5)	C41—P2—C51—C56	−164.3 (3)
P1—C11—C16—C15	−178.5 (3)	Pd1—P2—C51—C56	−35.9 (3)
C14—C15—C16—C11	1.9 (6)	C56—C51—C52—C53	0.9 (6)
C11—P1—C21—C22	−85.4 (3)	P2—C51—C52—C53	−178.3 (3)
C31—P1—C21—C22	166.5 (3)	C51—C52—C53—C54	−0.6 (6)
Pd1—P1—C21—C22	36.6 (3)	C52—C53—C54—C55	−0.3 (6)
C11—P1—C21—C26	93.3 (3)	C53—C54—C55—C56	0.7 (6)
C31—P1—C21—C26	−14.8 (3)	C54—C55—C56—C51	−0.3 (6)
Pd1—P1—C21—C26	−144.7 (3)	C52—C51—C56—C55	−0.5 (6)
C26—C21—C22—C23	−1.4 (6)	P2—C51—C56—C55	178.8 (3)
P1—C21—C22—C23	177.3 (3)	C51—P2—C61—C62	8.2 (3)
C21—C22—C23—C24	1.7 (7)	C41—P2—C61—C62	−102.4 (3)
C22—C23—C24—C25	−0.2 (7)	Pd1—P2—C61—C62	130.1 (3)
C23—C24—C25—C26	−1.5 (7)	C51—P2—C61—C66	−167.8 (3)
C24—C25—C26—C21	1.7 (6)	C41—P2—C61—C66	81.6 (3)
C22—C21—C26—C25	−0.3 (6)	Pd1—P2—C61—C66	−45.9 (3)
P1—C21—C26—C25	−179.0 (3)	C66—C61—C62—C63	−0.2 (6)
C11—P1—C31—C36	−170.3 (3)	P2—C61—C62—C63	−176.1 (3)
C21—P1—C31—C36	−61.2 (3)	C61—C62—C63—C64	0.2 (6)
Pd1—P1—C31—C36	60.9 (3)	C62—C63—C64—C65	−0.3 (6)
C11—P1—C31—C32	9.6 (3)	C63—C64—C65—C66	0.3 (6)
C21—P1—C31—C32	118.7 (3)	C64—C65—C66—C61	−0.3 (6)
Pd1—P1—C31—C32	−119.2 (3)	C62—C61—C66—C65	0.2 (5)
C36—C31—C32—C33	0.1 (5)	P2—C61—C66—C65	176.3 (3)
P1—C31—C32—C33	−179.7 (3)	C3^i^—C1—C2—C3	0.5 (10)
C31—C32—C33—C34	0.0 (6)	C1—C2—C3—C1^i^	−0.5 (10)

Symmetry code: (i) −*x*+1, −*y*+2, −*z*+1.
